# Supplementary material for: Effect of Oregano Essential Oil on Growth and Composition of Gut Prokaryote Microbiota on Striped Bass (Morone saxatilis)
Source: Microorganisms. 2025 Jan 25;13(2):264. doi: 10.3390/microorganisms13020264 (PMC11858431; doi:10.3390/microorganisms13020264)
Supplement: Supplementary file 1 [file microorganisms-13-00264-s001.zip › Figure S1_All_enriched_metabolic_pathways.pdf]

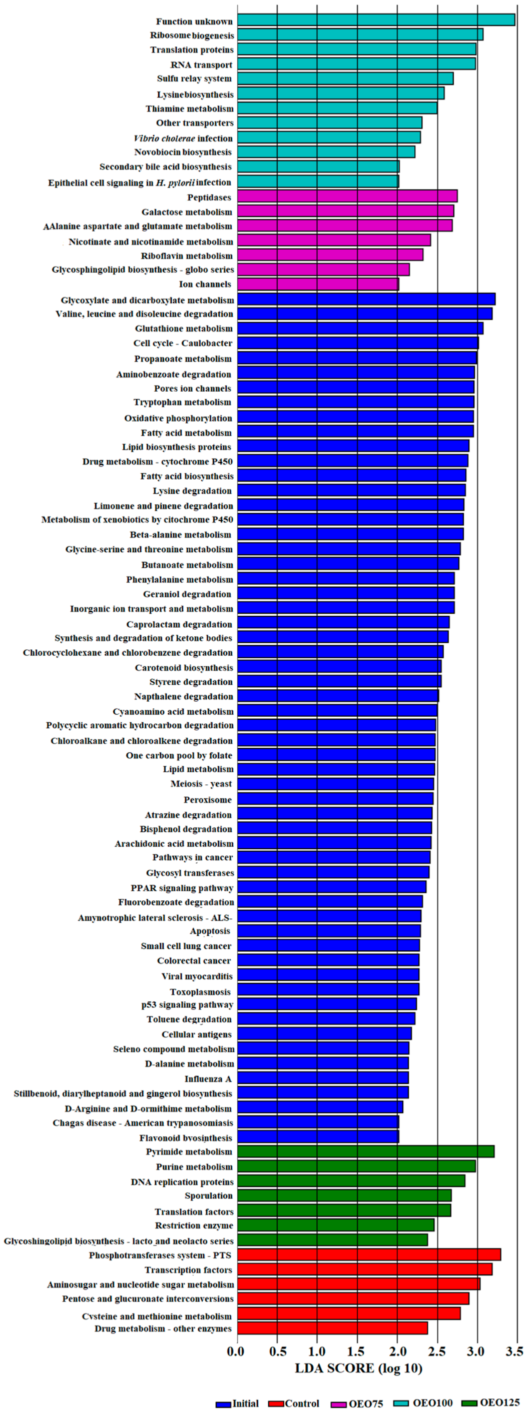

Figure S1. Complete enriched metabolic pathways in KEGG level 3 (class) predicted for the differentially expressed taxa in the gut microbiota of striped bass experimental groups (Control, OEO75, OEO100, and OEO125) and initial samples, using the LEFSe analysis and shown as log10 LDA score for each group. The image shows all the predicted functions of the initial samples' microbiota, which was the most enriched group.
