# Supplementary material for: Effect of Oregano Essential Oil on Growth and Composition of Gut Prokaryote Microbiota on Striped Bass (Morone saxatilis)
Source: Microorganisms. 2025 Jan 25;13(2):264. doi: 10.3390/microorganisms13020264 (PMC11858431; doi:10.3390/microorganisms13020264)
Supplement: Supplementary file 1 [file microorganisms-13-00264-s001.zip › Figures S2 and S3_Initial samples_microbiome_analysis.pdf]

Microbiome diversity analysis of initial samples

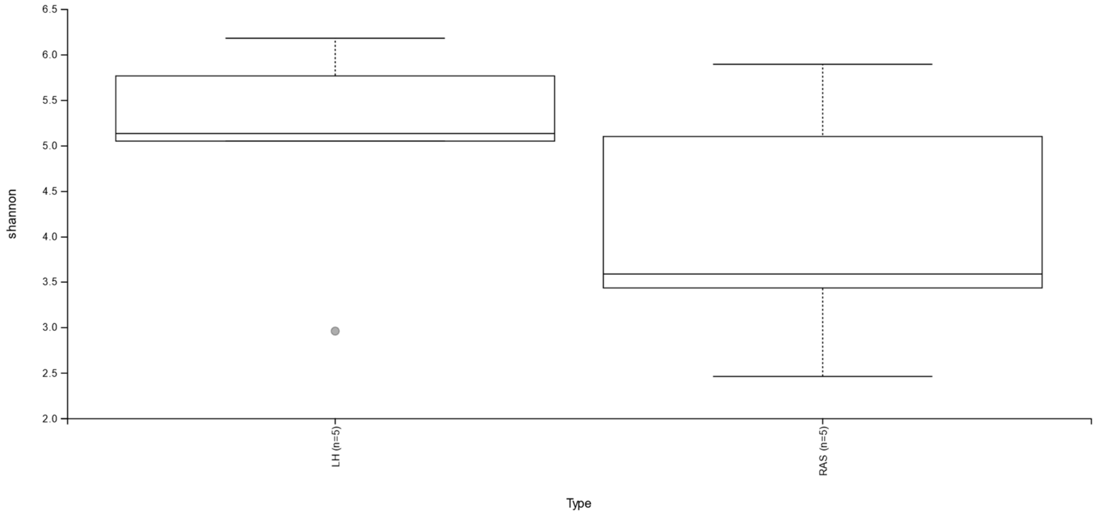

Figure S2. Box plot of Shannon index of the initial samples of seawater recirculation system (RAS) and flow-through system (LH) to assess richness. Shannon index (H= 0.88) tested by Kruskal-Wallis pairwise did not show differences in the Shannon index of the richness of the microbiome of both systems (p = 0.34).

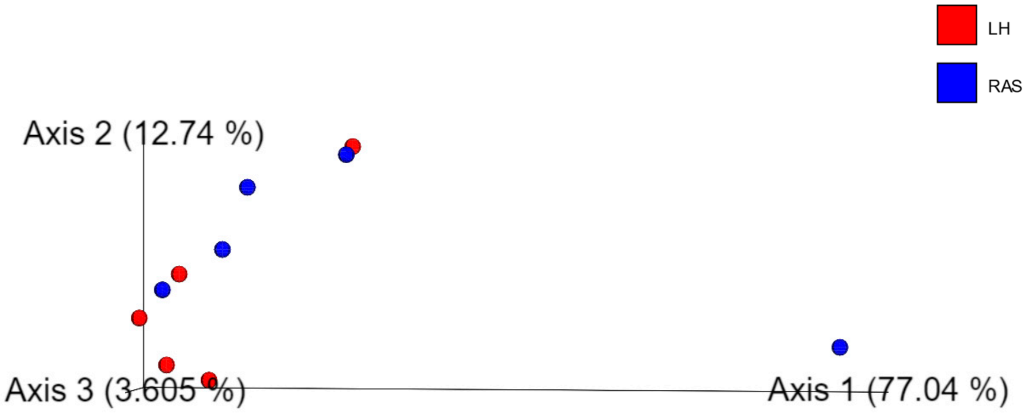

Figure S3. Principal coordinate analysis (PCoA) plot for beta diversity index of the initial samples of seawater recirculation system (RAS) and flow-through system (LH). The plot is based on the weighted phylogenetic distances of UniFrac, where one unique cluster with the components of both systems with one outlier is appreciated—no differences in the microbiome of both systems were detected by the PERMANOVA analysis (p=0.22).
